# An analytical framework for optimizing variant discovery from personal genomes

**DOI:** 10.1038/ncomms7275

**Published:** 2015-02-25

**Authors:** Gareth Highnam, Jason J. Wang, Dean Kusler, Justin Zook, Vinaya Vijayan, Nir Leibovich, David Mittelman

**Affiliations:** 1 Gene by Gene Ltd, Houston, Texas 77008, USA; 2 Biosystems and Biomaterials Division, National Institute of Standards and Technology, Gaithersburg, Maryland 20899, USA; 3 Virginia Bioinformatics Institute, Virginia Tech, Blacksburg, Virginia 24061, USA

## Abstract

The standardization and performance testing of analysis tools is a prerequisite to widespread adoption of genome-wide sequencing, particularly in the clinic. However, performance testing is currently complicated by the paucity of standards and comparison metrics, as well as by the heterogeneity in sequencing platforms, applications and protocols. Here we present the genome comparison and analytic testing (GCAT) platform to facilitate development of performance metrics and comparisons of analysis tools across these metrics. Performance is reported through interactive visualizations of benchmark and performance testing data, with support for data slicing and filtering. The platform is freely accessible at http://www.bioplanet.com/gcat.

## Abstract (web summary)

The standardization of clinical sequencing data generation and analysis is of critical importance. Here, the authors develop the Genome Comparison and Analytic Testing platform to facilitate the development of performance metrics and comparisons of analysis tools for clinical sequencing studies.

## Graphical abstract

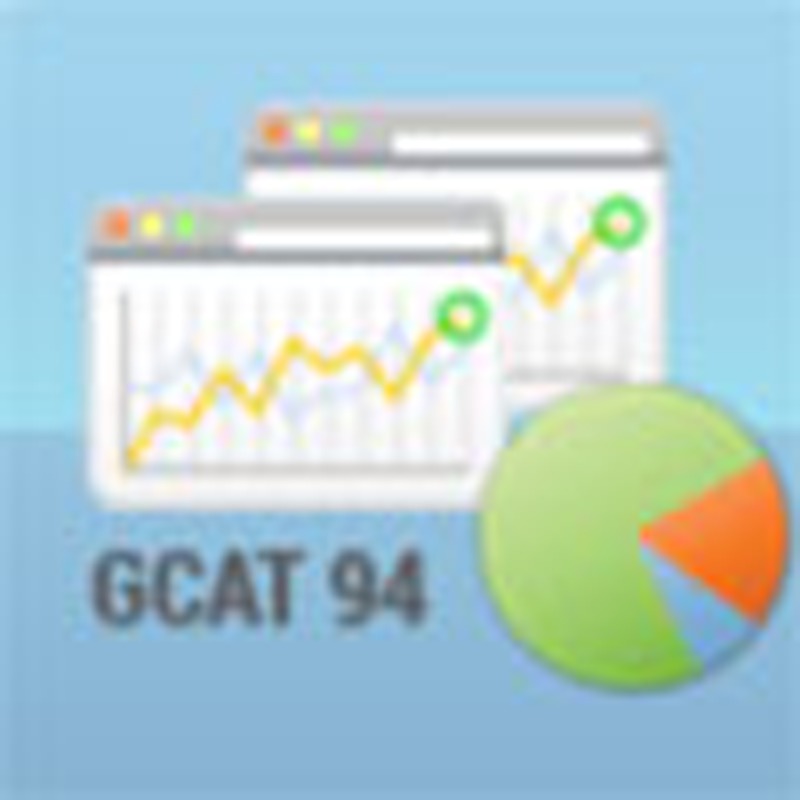

The recent affordability and throughput^1^ of next-generation sequencing technologies has enabled routine genome-wide sequencing at any scale^2^. As these new sequencing technologies penetrate the clinic, the bottlenecks are no longer around the amount of DNA sequence that can be screened; instead, they occur in the need for analysis methods for identifying and interpreting genetic variation^3^. The proper identification of genetic variation is a prerequisite for sensitive and accurate clinical tests and is heavily influenced by the technology platform^4^, sequencing assay^5^ and analysis method^6^,^7^. In the absence of perfectly described whole genomes, evaluating the performance of variant calling methods is not straightforward. Authors make valiant attempts to compare their tools to the state-of-the-art when they publish an update or new method, but the utilization of particular metrics and data sets can introduce bias into the performance test. Often, the comparisons are quickly obsolete, sometimes upon publication, because new tools and new versions of tools are available at such a regular frequency.

One way to address this challenge is to develop standard metrics and data sets for performance testing of genome analysis tools. Some groups such as the Genome in a Bottle (GIAB)^8^ consortium have developed highly confidence call sets that can be used as a proxy for truth sets. For the GIAB call set, the group produced a set of genotypes for the deeply sequenced NA12878 genome from the HapMap^9^ and 1,000 Genomes^10^ projects. These genotypes are an integration of 14 data sets from five sequencing platforms, seven read mappers and three variant callers. An orthogonal approach^11^ was recently described by Heng Li and uses the haploid CHM1 genome to estimate error from heterozygous calls. Defining performance and establishing standard metrics and data sets is critical for accelerating improvements to genome analysis tools^12^.

Here, we report the development of an open and collaborative platform for comparing analysis tools using various performance metrics and data sets. The genome comparison and analytic testing (GCAT) platform hosts raw sequence reads that users can download and operate on, using their own analysis pipelines. The user can then return the results of the pipeline to GCAT to benchmark the analysis and to compare it with other analysis pipelines applied to the same data sets. The benchmark results can be customized and shared with others.

## Results

The GCAT platform provides two kinds of benchmarks: an alignment test for evaluating short-read mappers and a variant calling test for evaluating germline single-nucleotide polymorphism (SNP) and indel variant callers. The alignment test is based on simulated reads with data sets for paired-end and single-end reads, read lengths from 100 to 400 bp and various mutation models. The variant calling test is based on sequencing data for the NA12878 genome that was generated using the Illumina, Ion Torrent and Ion Proton sequencing platforms (Supplementary Table 1). The GCAT user experience is summarized in Supplementary Fig. 1. In a typical workflow, the user downloads a simulated or actual data set as a FASTQ file and performs an analysis locally. The output of the analysis, a binary alignment map file for alignment testing or a variant calling format file for variant caller testing, is then uploaded to the GCAT site and the results are evaluated on the cloud. Without any coding or scripting, users can dynamically interact with the results, partition the data in various ways or customize the reporting/plotting of results. GCAT functions as a ‘data playground’, in which users can compare tools and then dive deep into the comparison to narrow in on benefits and limitations of various tools. The customized reports, plots and tables can be shared directly or through embedded links to the GCAT site posted to online communities such as SEQanswers^13^. In the remainder of this report, we highlight observations from alignment and variant calling benchmarks, and, as a demonstration of the utility of GCAT, we feature figures and data tables in this manuscript generated using the GCAT platform.

**Figure 1:**
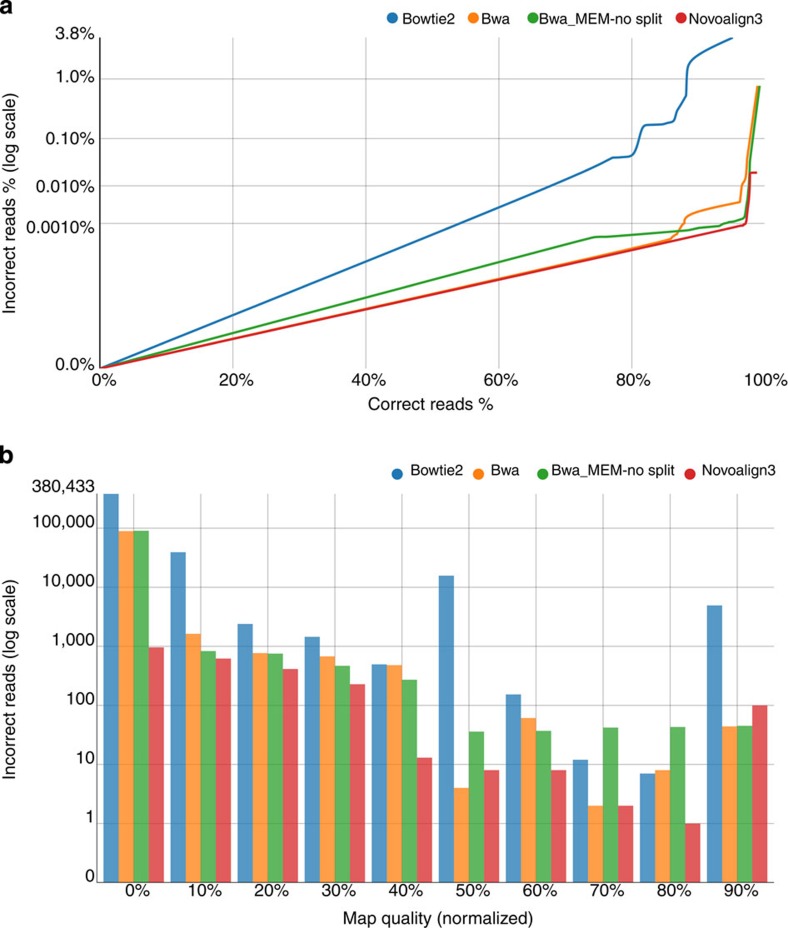
Benchmarking the accuracy of read alignments and the calibration of mapping quality scores. Mapping benchmarks were performed using simulated paired-end 100-bp Illumina reads. (**a**) The ROC-like curve illustrates, for each mapper, the number of incorrectly mapped reads as a function of correctly mapped reads, sorted by map quality. As such, greater accuracy is graphically represented as a lower curve that is farther right. Mapping quality thresholds begin at the highest quality and then progressively decrease. (**b**) To directly characterize mapping quality scores, a histogram indicates the distribution of incorrect reads across normalized mapping quality scores for various tools. Read count is displayed on a log scale, and mapping qualities are binned by 10%.

Mapping algorithms have continued to steadily improve, and in just the past year there have been major updates to Burrows–Wheeler alignment tool (BWA)^14^ and Novoalign (http://www.novocraft.com), two leading short-read mappers for the Illumina platform. Using 12 million simulated paired-end 100-bp Illumina reads, we benchmarked the recently released BWA-MEM (http://bio-bwa.sourceforge.net) and Novoalign3, against Bowtie2 (ref. 15) and BWA. The total number of mapped reads ranges from 95.19% (11,370,489) for Bowtie2 to 99.22% (11,814,790) in BWA-MEM. The mappers also differed in the number of incorrectly mapped reads, with Bowtie2 incorrectly mapping 3.72% (444,673), but with BWA and BWA-MEM incorrectly mapping 0.777% (92,854) and 0.779% (93,091), respectively. Novoalign3 made the fewest mapping mistakes with only 0.019% (2,194) reads mapped incorrectly (Supplementary Table 2). In Fig. 1a, a receiver-operating characteristic (ROC)-like curve illustrates, for each mapper, the number of incorrectly mapped reads as a function of correctly mapped reads, sorted by mapping quality. Novoalign3 leads in this comparison with 0.00092% of reads incorrectly when 97% of reads are mapped correctly. At the same percentage of correctly mapped reads, BWA-MEM incorrectly maps 0.0015% of reads, thus putting these two newer mappers at nearly the same accuracy, when considering mapping quality. We also find that using simulated paired-end 250-bp Illumina reads, the performance of the evaluated mappers ranks in the same order (Supplementary Fig. 2). The incorrect reads clustered generally cluster at low-complexity regions of the genome (Supplementary Fig. 3).

While assessing the number of correctly mapped reads is a key consideration in benchmarking short-read mappers, it is also important that a mapper properly assesses the confidence in mapped reads. Mapping quality scores can help identify suspect reads that might lead to less confidence in downstream variant calling steps. In Fig. 1b, we report mapping quality score percentiles for incorrectly mapped reads. For Novoalign3, 2,124 (96.8%) incorrect read alignments were assigned mapping quality scores in the lower 30% of quality scores and 52 (0.24%) of incorrect read alignments were in the top 20% of scores. While BWA-MEM incorrectly mapped a much greater number of reads, a similar proportion of reads were assigned low mapping quality scores. For BWA-MEM, 92,533 (98.9%) incorrect read alignments were assigned mapping quality scores in the bottom 30% of scores and 98 (0.001%) were assigned scores in the top 20%. For BWA, 91,580 (98.6%) of incorrect read alignments were assigned mapping quality scores in the bottom 30% and 52 (0.00056%) incorrectly aligned reads were assigned scores in the top 20%. Bowtie2 performed the worst, with 421,948 (94.9%) incorrectly aligned reads assigned scores in the bottom 30%, but 4,924 (0.011%) incorrectly mapped reads were assigned scores in the top 20%. Considering the above performance metrics, Novoalign3, followed by BWA-MEM, clearly outperform the older BWA and Bowtie2. The incredibly low number of reads incorrectly mapped by Novoalign3 comes at a cost of mapping fewer reads. Novoalign3 reports 137,819 (1.15%) reads as unmapped compared with BWA-MEM, which maps all but six (0.0001%) reads (Supplementary Table 2). Although Novoalign excels in mapping accuracy, BWA-MEM is very close in accuracy, and for applications where sensitivity is a primary concern BWA-MEM could be the better overall choice.

**Figure 2:**
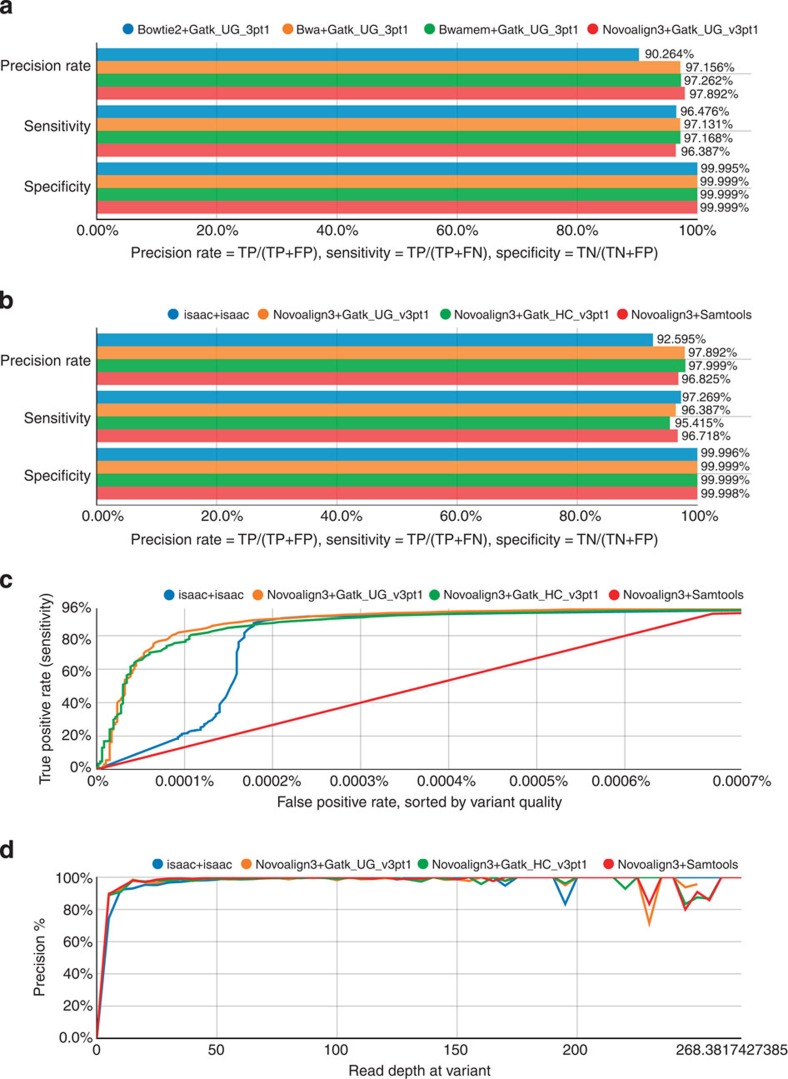
Performance testing variant callers. The Genome in a Bottle confident call set is used as the ‘ground truth’ for the NA12878 genome. Variant calling pipelines are evaluated based on their concordance to the confident call set in the high-confidence regions. (**a**) Precision, sensitivity and specificity metrics are shown for pipelines in which various mappers are used to generate the read alignments, but the same variant caller, GATK UnifiedGenotyper, is used to identify variants. (**b**) Precision, sensitivity and specificity metrics are shown for Illumina’s Isaac pipeline compared with three pipelines in which the same mapper, Novoalign3, was used to generate read alignments and different variant callers were used. (**c**) True-positive rate (TP/(TP+FN)) is plotted as a ROC-like curve and as a function of false-positive rate (FP/(FP+TN)), sorted by the variant quality score threshold. For each threshold, sites with variant quality scores above the given threshold are counted as true or false positives, and sites with variant quality scores below the given threshold are counted as true or false negatives. (**d**) Variant calling precision as a function of read depth for the different pipelines. The abbreviations ‘UG’ and ‘HC’ represent UnifiedGenotyper and HaplotypeCaller, respectively.

There are clear differences in how short-read mapping algorithms perform, and to assess the impact of these differences on variant calls, we constructed variant calling pipelines in which we varied the mapping algorithm but used the same variant caller. The mapping algorithm can impact variant calling in two ways: (1) incorrect general placement of the reads in the reference genome and (2) incorrect local alignment of the reads around indels and complex variants. One metric that GCAT leverages for variant caller benchmarking is the GIAB high-confidence call set^8^. While not completely free from bias, this call set allows for the enumeration of true-positive calls (TP), false-positive calls (FP) and false-negative calls (FN). We mapped 150 × Illumina data from exome capture of the NA12878 genome, using Novoalign3, BWA-MEM, BWA and Bowtie2, and then used GATK UnifiedGenotyper^16^ to identify variants. With this data set, users can determine the combined effect of mappers and variant callers on the accuracy of variant calls. Figure 2a plots precision (TP/(TP+FP)), sensitivity (TP/(TP+FN)) and specificity (TN/(TN+FP)) for the various pipelines. The Novoalign3-based pipeline produced the highest precision calls (97.89%), followed closely by BWA-MEM (97.26%), BWA (97.16%) and then Bowtie2 (90.26%). The precision of Novoalign3 comes at a cost of sensitivity. Novoalign3 featured a sensitivity of 96.39% compared with BWA-MEM (97.17%), BWA (97.16%) and Bowtie2 (96.48%). The loss in sensitivity comes from the reduced TP calls in the Novoalign3-based pipeline (20,806 calls) versus BWA-MEM (23,128 calls), BWA (23,126 calls) and Bowtie2 (22,945 calls). However, Novoalign3 does feature the lowest number of FP calls (Supplementary Table 3).

To assess the performance of popular variant callers, we constructed pipelines that utilized a common mapping algorithm, but a different variant calling tool. Using Novoalign3 as the mapper, we called variants using GATK HaplotypeCaller, GATK UnifiedGenotyper and Samtools^17^. We also compared these pipelines against Isaac^18^, which is a mapping and variant calling tool developed by Illumina. The GATK HaplotypeCaller pipeline offers the best precision (98.00%), followed closely by the GATK UnifiedGenotyper pipeline (97.89%) and then Samtools (96.83%). The Isaac pipeline, which features an integrated mapper and variant caller, had the worst precision (92.60%) (Fig. 2b). However, the Isaac pipeline featured the highest sensitivity (97.27%) compared with Samtools (96.72%), UnifiedGenotyper (96.39%) and HaplotypeCaller (95.42%).

Just as it is important that mappers properly score their alignments, the best variant callers must rank the confidence of their calls. This is typically done through the assignment of a variant quality score. In Fig. 2c, a ROC-like curve plots the true-positive rate as a function of false-positive rate, sorted by variant quality. GATK UnifiedGenotyper and GATK HaplotypeCaller score their calls such that there is great separation between TP and FP calls at high variant quality. Samtools and Isaac have weaker variant quality scores, demonstrated by the proportion of FP calls assigned a high variant quality score. Finally, Fig. 2d plots the relationship between precision and read depth. Here, the tools all perform similarly with precision optimized once read depth reaches about 30 × coverage.

## Discussion

In our benchmarking survey, we found that short-read mapping algorithms still continue to improve, and that these improvements affect the accuracy of read alignments and the precision and sensitivity of variant calls. We find that variant callers also differ in performance, even when operating on the same read alignments. Tools also differed in their ability to score poor alignments or poor variant calls. It is worth noting that as variant callers improve, it will be increasingly important to calibrate variant quality scores so that sensitivity can be maximized with a minimum detriment to precision. In particular, as variant callers become more sensitive, it will be increasingly important to fine-tune variant quality scores to maintain precision through the filtering of variant quality scores. Furthermore, benchmarking on exome data likely overestimates the performance of analysis methods for whole genomes. The future use of whole genomes and multiple samples will help improve performance measurements. The GCAT platform was created to help developers and end-users benchmark and optimize analysis pipelines. GCAT is powerful because it enables the dynamic comparison of emerging tools, as well as variations and updates to existing pipelines. Users can make comparisons with standardized metrics and data sets, interactively digging into the comparisons to stratify the results using parameters such as sequencing depth, quality score and mutation class. GCAT enables direct sharing of results with others or embedding of results online, and the tool can be used in an unrestricted manner by anyone. Since launching in April 2013, GCAT has amassed over 200,000 visitors from 194 countries and now hosts more than 2,500 benchmark reports. We plan to continue developing GCAT, including working with the new Global Alliance for Genomic Health (http://genomicsandhealth.org) Benchmarking working group to implement a graphical interface to the standard performance metrics and benchmarking tools being developed by the group. It is our hope that the resource will help drive the discussion on reference materials and performance testing, and help grow the adoption of genome-wide sequencing in the clinic.

## Methods

### GCAT report generation

Reference data sets were downloaded from GCAT, processed on local infrastructure and then the binary alignment map and variant calling format files were returned to GCAT where benchmarking reports were generated. The reports used to build Figs 1 and 2 in the main paper are shown below:

http://www.bioplanet.com/gcat/reports/23/alignment/100bp-pe-small-indel/bowtie2/compare-18-22-200 (for Fig. 1)

http://www.bioplanet.com/gcat/reports/2305/variant-calls/illumina-100bp-pe-exome-150x/bowtie2-gatk-ug-3pt1/compare-2303-2304-2788/group-read-depth and

http://www.bioplanet.com/gcat/reports/530/variant-calls/illumina-100bp-pe-exome-150x/isaac-isaac/compare-2850-2788-2851/group-read-depth for Fig. 2.

### Generating simulated read alignments

Chromosome 19 from build hg19 of the human reference sequence was used to generate simulated paired- and single-end reads for Illumina FASTQ data under several different mutation and read length parameters with the short-read simulator, DWGSIM v0.1.11 (https://github.com/nh13/DWGSIM). These simulated data sets feature read lengths of 100, 150, 250 and 400 bp (parameters -1 <read length> and -2 <read length>), with a 500-bp insert (50 bp s.d.) for paired-end libraries. Small 1–10-bp indels and large 10–24-bp (–I 10) indels occurred in 10% (–R 0.1) of mutations with a 0.1% (–r 0.001) chance of mutation occurrence. The number of reads generated is dictated by the specification of 20 × coverage (–C 20) of chromosome 19. A simulation of a single smaller chromosome is unlikely to capture the complete spectrum of sequence structure and complexity found throughout the genome, but serves as a reasonable surrogate for distinguishing the performance of short-read mapping algorithms. We find that a whole-genome simulation with similar parameters (changed –C 15 for 15 × coverage) reflects similar differences between algorithms as does the simulated chromosome 19 data used by GCAT. We also tried a second simulator, ART (http://www.niehs.nih.gov/research/resources/software/biostatistics/art/), and found that the results were consistent with evaluations based on simulated data produced by DWGSIM (Supplementary Table 4).

### Running variant calling pipelines

Where possible, all tools were run with default settings. To compensate for the small number of secondary alignments that are produced by split reads in BWA-MEM default mode, the –M parameter was used to suppress this operation. This ensured a more fair comparison with other mappers that produce only primary alignments. The use of the –M parameter does not, however, significantly affect BWA-MEM results compared with the default or the other mappers. For the variant calling analysis, where pipelines using BWAMEM were run on real exome data, we returned to using BWA-MEM default settings. Samtools variant calling was executed with the recommended pipe to ‘bcftools view -bvcg-><out>.bcf; bcftools view <out>.bcf | vcfutils.pl varFilter -D100><out>.flt.vcf’. The iSAAC alignment was run with ‘--keep-unaligned’, ‘--realign-gaps yes’ and iSAAC variant calling step used the build’s provided config file found in ${INSTALL_ROOT}/etc/. The tool versions are as follows: Bowtie2 v2.0.0-beta5, GATK v3.1-1-g07a4bf8, Samtools v0.1.18, BWA v0.7.5a-r405, Novoalign v3.00.04, and iSAAC v01.13.06.20.

### Benchmarking variant calling

The second major component of GCAT allows comparison of variant calls from different methods and performance assessment of individual variant call sets against ‘ground truth’ sets. The ‘ground truth’ sets currently used in GCAT are SNP sites genotyped by a microarray and a set of high-confidence SNP, indel and homozygous reference genotypes developed for NA12878 by NIST and the GIAB Consortium, version 2.18. While neither of these data sets is perfectly accurate or comprehensive, both can provide estimates of sensitivity, specificity and precision rate, as well as ROC-like curves. To elaborate on specificity in particular, we count as true negatives every base in the high-confidence regions that is not covered by a variant in the benchmark or in the test set. In general, this is very close to the total number of bases in the high-confidence regions, because most bases are homozygous reference. Therefore, specificity is almost always very close to 100%, and precision rate may be a more useful statistic in most cases. We have focused performance estimation on the exome in this work, because the exome is well studied for clinical and functional applications. Additional data sets and entire genomes are planned additions for future iterations of GCAT. We decided against benchmarking variant calls with simulated data sets due to challenges in realistically modelling them.

The detection methodology for microarrays is different from sequencing, so it can be useful as an orthogonal way to assess accuracy of sequencing. However, microarrays contain only known variants for which probes are designed for, which tend to be in regions of the genome that are easier to sequence. In addition, microarrays can give incorrect results due to various technical challenges including instances where nearby phased variants interfere with probe binding.

To assess a greater number of variants, including indels, GCAT also allows users to benchmark their analysis using the GIAB high-confidence genotypes for NA12878. These calls were generated by integrating 14 whole-genome and exome data sets from five different sequencing technologies. When data sets yielded discordant genotype calls, characteristics of bias (for example, strand bias and clipping of reads) were used to arbitrate between data sets. The GIAB high-confidence genotype calls contain 23,625 SNPs, 562 indels and 46,468,537 homozygous reference positions in the exome. For comparison of variant calls with the GIAB calls, GCAT excludes any variants at positions where GIAB does not make a high-confidence genotype call. The GIAB calls contain more difficult regions than the microarrays, but they still exclude 22.6% of the genome. The excluded regions include regions difficult to call accurately using short-read next-generation sequencing, such as regions with possible structural variants, regions with low mapping quality or coverage, simple repeats, known segmental duplications and sites where discordant genotypes between data sets could not be resolved. In addition, complex variants (nearby SNPs and indels) are difficult to assess because different mappers and variant callers will represent them differently. Therefore, any 10 base regions that contain an indel and another variant in the GIAB call set are excluded from the comparison on GCAT. By comparing variant calls from different mappers and variant callers with the GIAB call set on GCAT, the user can learn the strengths and weaknesses of each method.

## Author contributions

J.J.W., D.K., N.L. and D.M. developed the GCAT platform. G.H., J.J.W., J.Z. and D.M. designed the experiments. G.H., J.J.W., V.V., J.Z. and D.M. performed the experiments. G.H., J.Z. and D.M. wrote the manuscript.

## Additional information

**How to cite this article:** Highnam, G. *et al*. An analytical framework for optimizing variant discovery from personal genomes. *Nat. Commun.* 6:6275 doi: 10.1038/ncomms7275 (2015).

## Supplementary Material

Supplementary Information Supplementary Figures 1-3, Supplementary Tables 1-4 and Supplementary Reference
