# Supplementary material for: An analytical framework for optimizing variant discovery from personal genomes
Source: Nat Commun. 2015 Feb 25;6:6275. doi: 10.1038/ncomms7275 (PMC4351570; doi:10.1038/ncomms7275)
Supplement: Supplementary Information — Supplementary Figures 1-3, Supplementary Tables 1-4 and Supplementary Reference [file ncomms7275-s1.pdf]

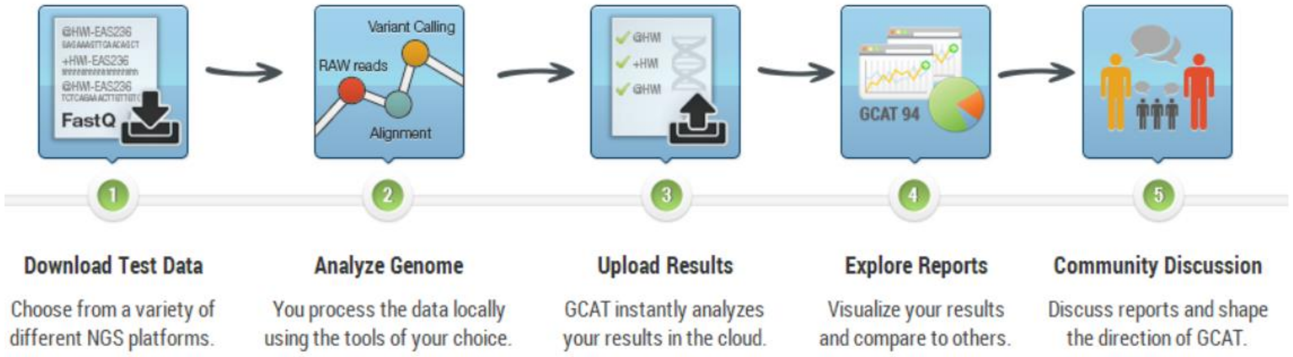

Supplementary Figure 1. The GCAT user experience.
Users download simulated or real GCAT data files, execute a pipeline of their choice (locally), return the results to GCAT, and then view an interactive report that benchmarks the performance of the pipeline.

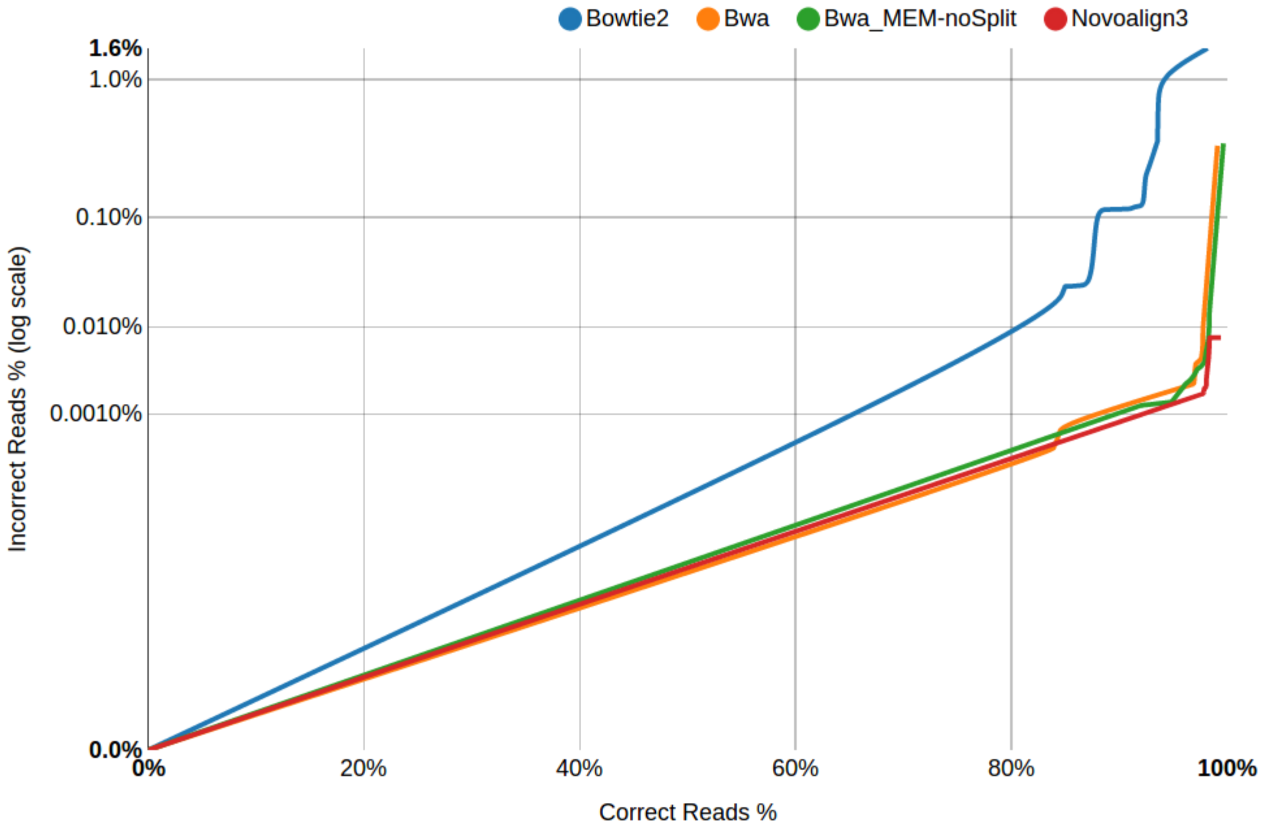

Supplementary Figure 2.Alignment accuracy for short read mapping algorithms
A ROC-like curve that shows incorrect alignmentsas a function of correct alignments, sorted by mapping quality score,for simulated 250bp paired end Illumina reads.

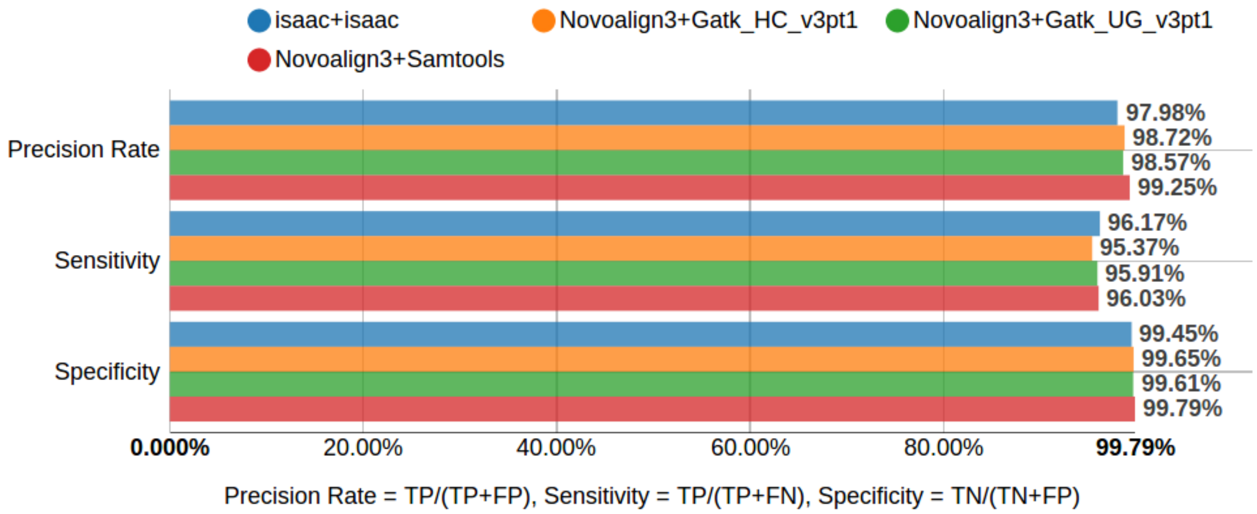

Supplementary Figure 3. SNP performance testing calculated from Illumina HumanOmni2.5-8v1 Array

Like the GIB call set, NA12878 genotypes from the Illumina HumanOmni2.5-8v1 Array can be used as a high confidence “ground truth” to measure precision, sensitivity, and specificity for variant calling pipelines. Pipeline names are in the format of mapper+variant caller so, for example, “Novoalign3+Samtools” indicates the reads were mapped with Novoalign3 and variants were identified in the resulting read alignments using Samtools.

Supplementary Table 1. Simulated and real GCAT data files for use in benchmarking.

**Simulations**			**Real Exome Data**			
Library	*Read Length*	*Mutation Type*	*Platform*	*Coverage*	*Read Type*	*Read Length (bp)*
Paired End	100	Small indels	Ion Torrent	20x (Ion Torrent)	Single End (Ion Torrent)	225 (Ion Proton)
Single End	150	Large indels	Illumina	150x (Illumina)	Paired End (Illumina)	100 (Illumina)
	250					
	400					

Supplementary Table 2. The counts of total reads in the simulated 100bp paired end Illumina library, correct alignments, incorrect alignments, and unmapped alignments for each short-read mapper.

Name	Total Reads	Total Correct	Incorrectly Mapped	Unmapped
**Bowtie2**	11,945,250	11,370,489 (95.19%)	444,673 (3.72%)	130,088 (1.09%)
**BWA**	11,945,250	11,814,790 (98.91%)	92,854 (0.78%)	37,606 (0.31%)
**BWA-MEM**	11,945,250	11,852,153 (99.22%)	93,091 (0.78%)	6 (0.0001%)
**Novoalign3**	11,945,250	11,805,078 (98.83%)	2,353 (0.020%)	137,819 (1.15%)

Supplementary Table 3. Performance testing of pipelines using the Genome-in-Bottle calls as “ground truth”.False positive calls are broken down into further categories: heterozygous variant instead of homozygous reference (Het-Ref), heterozygous variant instead of homozygous variant (Het-HomVar), homozygous variant instead of heterozygous variant (HomVar-Het), and homozygous variant instead of homozygous reference (HomVar-Ref). Pipeline names are in the format of mapper+variant caller so, for example, “Novoalign3+Samtools” indicates the reads were mapped with Novoalign3 and variants were identified in the resulting read alignments using Samtools.

Pipeline	True Positive	False Positive	True Negative	False Negative	Het-Ref	Het-HomVar	HomVar-Het	HomVar-Ref
**Bowtie2 +Gatk_UG_3pt1**	22,945	2,475	46,466,062	838	2,043	387	24	15
**Bwa +Gatk_UG_3pt1**	23,126	677	46,467,860	683	272	360	27	12
**Bwa_MEM +Gatk_UG_3pt1**	23,128	651	46,467,886	674	249	360	24	11
**Novoalign3 +Gatk_UG_v3pt1**	20,806	448	46,468,089	780	139	278	23	5
**isaac+isaac**	23,047	1,843	46,466,694	647	1,307	463	24	42
**Novoalign3 +Gatk_HC_v3pt1**	22,828	466	46,468,071	1,097	201	254	9	2
**Novoalign3 +Samtools**	22,842	749	46,467,788	775	166	149	28	12

Supplementary Table 4. Alignment tool performance testing between GCAT chr19 simulations and whole genome simulations from both DWGSIM and ART. For BWA and Bowtie2, the alignment accuracy statistics are compared between GCAT's chr19 simulations (GCAT) and whole genome simulation made with similar parameters in DWGSIM (WGS). Additionally, chr19 and chr21 simulations with the ART simulator (ART and chr21 respectively) [1] were generated using the non-default parameters for read length 100bp (-l 100), 10x coverage (-f 10), 700bp fragment size (-m 700), and 50bp standard deviation (-s 50). Although the performance of the tools on WGS simulations is worse than forchr19, the accuracy of the tools in relation to each other is still the same. Likewise, the ART chr19 simulations produced reads that were more difficult to map than the GCAT chr19 DWGSIM data, but did not change the order of the tools when sorted by performance. The drop in accuracy for alignment of reads from WGS data is likely due to the larger numbers of repetitive regions across the whole genome that might not be well represented in chr19.

Method	Total Reads	Correctly Mapped (%)	Incorrectly Mapped (%)	Unmapped (%)
**WGS BWA-MEM**	495,341,256	456,828,469 (92.22)	11,382,591 (2.30)	27,130,196 (5.48)
**WGS Bowtie2**	495,341,256	443,365,818 (89.51)	20,875,906 (4.21)	31,099,532 (6.28)
**GCAT BWA-MEM**	11,945,250	11,852,153 (99.22)	93,091 (0.78)	6 (0.000050)
**GCAT Bowtie2**	11,945,250	11,370,489 (95.19)	444,673 (3.72)	130,088 (1.09)
**ART BWA-MEM**	5,581,122	5,421,159 (97.13)	124,053 (2.22)	35,910 (0.64)
**ART Bowtie2**	5,581,122	4,059,365 (72.73)	263,567 (4.72)	1,258,190 (22.54)
**chr21 BWA-MEM**	3,508,754	3,422,731 (97.54)	66,830 (1.90)	19,193 (0.55)
**chr21 Bowtie2**	3,508,754	2,677,891 (76.32)	124,911 (3.56)	705,952 (20.11)

Supplementary References

1 Huang, W. *et al*. ART: a next-generation sequencing read simulator. *Bioinformatics***28**, 593-594, doi: 10.1093/bioinformatics/btr708 (2012).
